# Invasive bacterial infections in Gambians with sickle cell anemia in an era of widespread pneumococcal and hemophilus influenzae type b vaccination

**DOI:** 10.1097/MD.0000000000005512

**Published:** 2016-12-09

**Authors:** Germander Soothill, Saffiatou Darboe, Gibril Bah, Lawal Bolarinde, Aubrey Cunnington, Suzanne T. Anderson

**Affiliations:** a MRC Unit The Gambia, Atlantic Boulevard, Fajara, The Gambia; b Royal Free Hospital, Pond St, London, UK; c Section of Paediatrics, Department of Medicine, Imperial College London, London, UK.

**Keywords:** antimicrobial prophylaxis, bacteremia, sickle cell anemia, West Africa

## Abstract

There is relatively little data on the etiology of bacterial infections in patients with sickle cell anemia (SCA) in West Africa, and no data from countries that have implemented conjugate vaccines against both *Streptococcus pneumoniae* and *Haemophilus influenzae* type b (Hib).

We conducted a retrospective analysis of SCA patients admitted to the Medical Research Council Unit, The Gambia, during a 5-year period when there was high coverage of Hib and Pneumococcal conjugate vaccination. We evaluated 161 admissions of 126 patients between April 2010 and April 2015.

Pathogenic bacteria were identified in blood cultures from 11 of the 131 admissions that had cultures taken (8.4%, 95% CI 4.5–14.1%). The most frequent isolate was *Salmonella* Typhimurium (6/11; 54.5%), followed by *Staphylococcus aureus* (2/11; 18.2%) and other enteric Gram-negative pathogens (2/11; 18.2%) and there was 1 case of *H influenzae* non-type b bacteremia (1/11; 9.1%). There were no episodes of bacteremia caused by *S pneumoniae* or Hib.

The low prevalence of *S pneumoniae* and Hib and the predominance of nontyphoidal *Salmonella* as a cause of bacteremia suggest the need to reconsider optimal antimicrobial prophylaxis and the empirical treatment regimens for patients with SCA.

## 1 Introduction

Patients with sickle cell anemia (SCA) are at increased risk of bacterial infections, which are leading causes of morbidity and mortality, especially in populations that lack effective prophylaxis and treatment. Although >80% of people with SCA are born in Africa, paradoxically there is relatively little high quality data on infectious complications in these populations. Historically, studies from resource-rich settings have identified *Streptococcus pneumoniae, Haemophilus influenzae* type b (Hib), and nontyphoidal *Salmonella* (NTS) species to be the most common invasive bacteria.^[1–3]^ Studies from Africa have produced inconsistent results, with a large study from Kenya finding similar pathogens to those seen in western populations,^[4]^ whereas studies from Uganda, Nigeria, and more recently Tanzania, showing that *Staphylococcus aureus*, NTS species, and other enteric Gram-negative pathogens predominated.^[5–7]^ There are limited data about types of pathogens and efficacy of antimicrobial prophylaxis in West Africa and no published data from SCA patients in The Gambia.

Penicillin prophylaxis and conjugate vaccines against *S pneumoniae* and Hib have significantly improved prognosis and almost eliminated the additional risk of bacteremia associated with SCA in resource-rich settings.^[8–12]^ However, these are not routinely implemented in much of West Africa, despite the greatest global burden of disease.^[13]^ The Gambia is relatively unique in that Hib and pneumococcal conjugate vaccines are well established as part of the Expanded Program on Immunization.^[14]^ The 7-valent pneumococcal conjugate vaccine (PCV7) was introduced in 2009, followed by the 13-valent vaccine (PCV13) in 2011^[15]^ and the estimated proportion of Gambian infants receiving all 3 pentavalent vaccines (containing Hib) and PCV 13 is 89.3%.^[16]^

Based on the pathogen distribution found in recent studies, some commentators have challenged the benefits of penicillin prophylaxis in African populations.^[5]^ We aimed to review the predominant pathogens causing invasive bacterial infections in SCA patients admitted to the Medical Research Council Gambia (MRCG) Unit clinical wards through retrospective analysis of patient and laboratory records.

## 2 Methods

### 2.1 Setting and population

This study was conducted at the Clinical Services Department (CSD) of the MRCG, located in the urban, western coastal region where malaria transmission is relatively low.^[17]^ The Gambia is a small country (population 1.8 million in 2013) situated north of the equator in West Africa. National prophylaxis guidance for SCA recommends pyrimethamine (for malaria) and phenoxymethylpenicillin.

The CSD at the MRCG comprises an outpatient medical clinic seeing around 50,000 patients a year and a 42-bed inpatient unit. All patients with history or clinical findings suggestive of SCA are screened using a metabisulfite test, followed (if positive) by hemoglobin electrophoresis. There is no specific protocol for management of SCA complications; however, blood cultures are drawn from all patients with clinical features compatible with invasive bacterial infection. Other samples from sterile sites (e.g., cerebrospinal fluid [CSF]) are collected when clinically indicated. A high-quality, clinical diagnostic laboratory is located at the CSD and provides round-the-clock microbiology, hematology, and biochemistry services. Empirical antibiotic treatment for suspected sepsis is ampicillin and gentamicin.

### 2.2 Ethics

Ethical review was not required for this study since it was undertaken as a clinical audit to establish the prevalence and causes of invasive bacterial infections in SCA patients at the MRCG CSD.

### 2.3 Data collection

The admission records of all patients presenting to the CSD over a 5-year period (7th April 2010 to 7th April 2015) were reviewed. Patients with SCA were identified from discharge diagnosis records. We recorded whether patients were known to have SCA, what medications were taken prior to admission, hematological values, temperature, duration of admission, clinical diagnosis, bacteremia or other positive sterile site culture, and sensitivities of cultured pathogens.

We defined wet season as June to November and dry season as December to May. Severe anemia was defined as hemoglobin <50 g/L. For cultural and practical reasons, all patients admitted have axillary temperature measured, and for the purpose of this study, a fever cut-off value of ≥37.5°C was selected, which has been shown to increase sensitivity for predicting infection without greatly impacting specificity.^[18]^

### 2.4 Laboratory methods

SCA was diagnosed using hemoglobin electrophoresis. Full blood count analysis for each patient's sample was performed using either the automated Medonic M Series, 3 part differential Haematology Analyzer or the automated Cell DYN 3700, and 5 part differential Haematology Analyzer.

Blood cultures were performed using the BACTEC 9050 automated culture system. In total, 1 to 3 mL blood was inoculated into BD BACTEC PEDS PLUS/F culture for children and 3–10 mL in each aerobic and anaerobic vial for adults. Bottles were usually placed in the incubator within 2 hours of collection. If a delay occurred, bottles were pre-incubated at 35–37°C. Microbiological procedures were performed using standard media if bottles gave a positive signal within 5 days, after which they were reported negative. Further identification was done by cultural morphology, Gram staining, biochemical test kits, and serological agglutination. Staphylococcus isolates were identified by coagulase, mannitol fermentation, and Catalase tests. *Salmonella* Typhimurium was identified using the bioMerieux analytical profile index; API 20 E (Becton Dickinson, Sparks, MD), and characterized by serotyping using Statens Serum Institute *Salmonella* Sero-Quick kit (groups A-G) and *Salmonella* Sero-Quick ID kit (specified for Typhimurium and Enteritidis). Other enteric Gram-negatives were identified using the bioMerieux analytical profile index: API 20 E. Antibiotic susceptibility was assessed according to CLSI interpretation guide. For the purpose of this study, all organisms found as normal skin or oral flora were considered to be contaminants, including coagulase-negative *Staphylococci*, alpha-hemolytic *Streptococci* (other than *S pneumoniae*), and diphtheroids.

### 2.5 Statistical methods

Each hospitalization was considered as a separate event, although some patients were admitted more than once. Results are reported as proportions of total number of admissions, blood cultures or isolates, or as median values and interquartile ranges for quantitative variables. Where appropriate, the “binom” package in R was used to calculate Jeffrey's 95% confidence intervals for proportions. The Mann–Whitney *U* test was used to compare the hematological parameters of patients with and without a proven invasive bacterial infection. *P* value of <0.05 was used to define a significant difference.

## 3 Results

**Figure 1:**
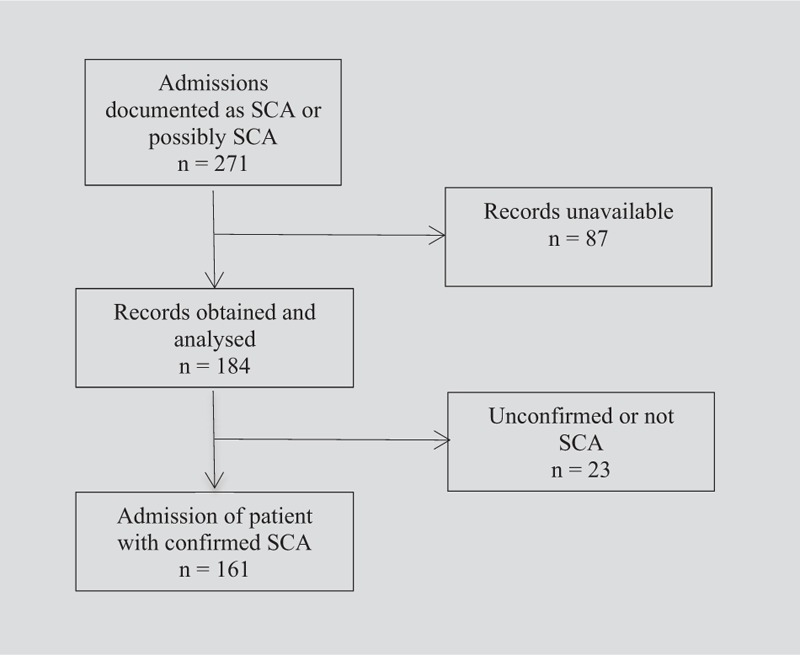
Flow diagram of case identification pathway. Vertical arrows indicate the flow of patients included in the data analysis; horizontal arrows indicate the patients who were excluded.

Data from 161 admission episodes (of 126 patients) with SCA were included in the analysis (Fig. 1). A summary of characteristics is shown in Table 1. Of 25 patients with >1 admission, the median time between discharge and readmission was 87 days and the minimum interval was 1 day. Five patients were readmitted within 1 week of discharge; no culture positive patients were recounted for the same bacterial infection. One patient had 2 admissions with positive blood cultures 1 month apart, *S aureus* was cultured on their first admission and *H influenzae* non-type b on their second; they had a negative HIV test. The median length of admission was 5 days, and there was 1 death of a patient diagnosed with an aplastic crisis with negative blood cultures. The median age of patients was 5 years (interquartile range [IQR]: 2–13 years). Just under half of patients had a new diagnosis of SCA made on admission (median age 4 years [IQR: 2–8 years]). There was no statistically significant difference in the hematological parameters of patients with and without a proven invasive bacterial infection (Table 2).

**Table 1:**
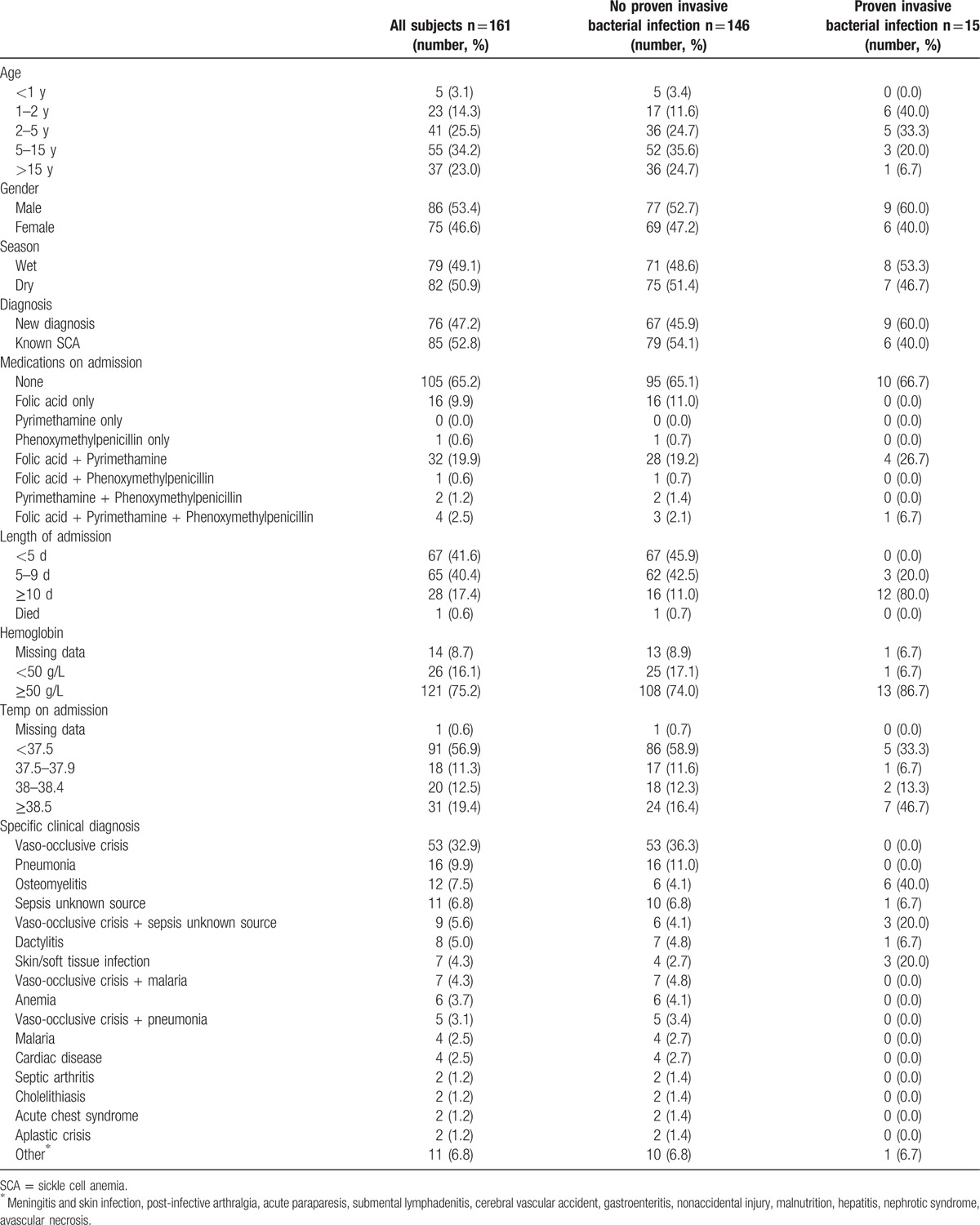
Patients’ characteristics by the presence of invasive bacterial infection.

**Table 2:**
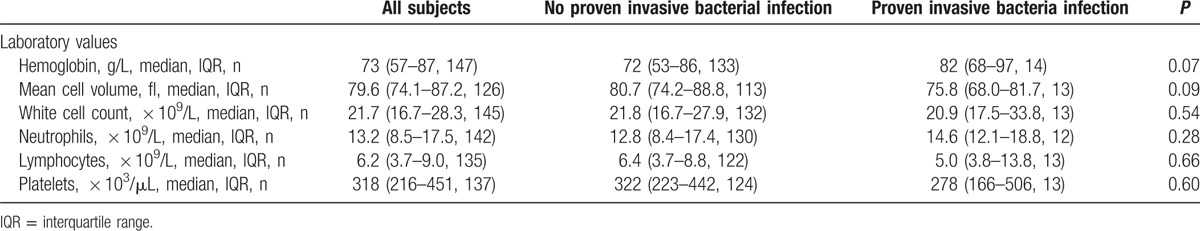
Comparison of patients’ hematological values on admission by the presence of invasive bacterial infection (using the Mann–Whitney *U* test no statistically significant difference for any parameters).

**Figure 2:**
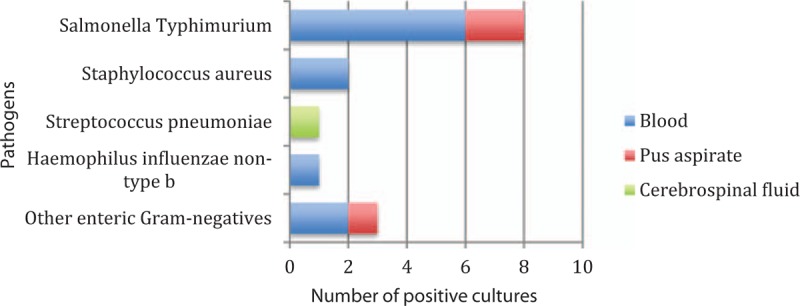
Pathogens identified from sterile site culture.

Of the 161 admission episodes, 131 (81%) had blood cultures taken, and 16 (12.2%, 95% CI 7.4–18.6%) of these were positive. Five blood cultures (3.8%, 95% CI 1.5–8.2%) grew suspected contaminants and 11 (8.4%, 95% CI 4.5–14.1%) yielded pathogenic organisms. The most frequent pathogenic isolates were NTS (6/11; 54.5%), other enteric Gram-negatives (2/11; 18.2%), and *S aureus* (2/11; 18.2%). No episodes of *S pneumoniae* or Hib bacteremia were identified (0/131, 0%, 95% CI 0–1.5%), but there was 1 case of *H influenzae* nontype b. 64% (7/11) of positive blood cultures occurred in children aged under 5. Cultures from other sterile sites (CSF and pus aspirates) were also analyzed, revealing 4 additional positive cultures (Fig. 2). One child with a new diagnosis of SCA, unknown immunization status, and not taking penicillin prophylaxis, had *S pneumoniae* isolated from CSF.

## 4 Discussion

SCA is estimated to be one of the biggest causes of premature death in West Africa, mainly attributed to infections.^[19]^ The prevalence of SCA in The Gambia is unknown, and the lack of prevalence data and the failure of children to be routinely screened either at birth or on hospital admission means that it is likely to be significantly underestimated. Four small cross-sectional surveys completed in rural Gambia estimated the percentage of newborns with SCA to be between 0.8% and 1.2%, with a high excess mortality in early childhood.^[20,21]^

Knowledge of the most common pathogens infecting patients with SCA could be used to improve antimicrobial prophylaxis and empirical treatment of infections. Variations in the prevalence of *S pneumoniae* and Hib in studies of bacteremia in SCA patients in Africa have been the cause of much debate.^[22]^ In this study, we found no cases of *S pneumoniae* or Hib bacteremia. One case of *S pneumoniae* was identified from CSF culture, and there was 1 case of *H influenzae* non-type b bacteremia. *S pneumoniae* and Hib infections are more common among younger SCA patients, and it has been suggested that studies of known SCA patients in sub-Saharan Africa are missing infections occurring in younger children due to late diagnosis of SCA.^[4]^ Although there are regional screening programs for SCA in sub-Saharan Africa,^[23]^ their coverage is minimal and diagnosis is usually made when a complication occurs. The median age in our study was 5 years, whereas that of children diagnosed on presentation (just under half of subjects) was 4 years, so it is possible that the low rates of *S pneumoniae* and Hib in this study are a reflection of this older age range and survivorship bias.

The Gambia has a well-established vaccination program, ahead of most other countries in sub-Saharan Africa.^[14–16]^ Therefore, it is highly likely that the prevalence of *S pneumoniae* and Hib infections is lower than other countries. A study conducted at MRCG before introduction of the PCV (2003–2005) found that *S pneumoniae* accounted for 45.2% of community acquired bacteremia in children, followed by *S aureus* (18.3%), *E coli* (9.7%), and NTS (8.6%).^[24]^ In contrast, a review of causes of bacteremia in the same unit after the establishment of these vaccination programs (2010–2014) showed a decline in *S pneumoniae* to just 15.1% with *S aureus* the predominant pathogen accounting for 24% of isolates (S. Anderson, unpublished data). This trend is mirrored by recent data from elsewhere in The Gambia.^[25]^

It is unlikely that the low yield of *S pneumoniae* observed in this cohort of patients is due to inadequate culture facilities. In contrast to many other low-resource settings, the MRCG has excellent clinical facilities and a co-located diagnostic microbiology laboratory. However, because this was a retrospective study it was not possible to determine time from blood collection to incubation, or the volume of blood collected for culture—both known to impact on culture yield and likelihood of contaminants.^[26]^ Nonetheless, the rate of blood culture contamination (3.8% of blood cultures taken and 31% of positive blood cultures) is lower than other studies in sub-Sahara Africa,^[7,27]^ and suggests poor technique is unlikely to have greatly influenced our results.

The retrospective nature of the study means we did not have some information that could be important for interpreting our results (immunization status; clinical justification for taking blood cultures; the decision making process used to assign a final diagnosis; or the serotype of the pneumococcal meningitis isolate). We also do not have denominator or pre-vaccination data. We do not know for certain about prior antimicrobial treatment, which may have resulted in negative culture results. However, only 5% of patients were documented as taking phenoxymethylpenicillin prophylaxis on admission, suggesting this effect may be small. Rates of bacteremia may also have been underestimated because blood cultures were not performed on all patients. However, our results are consistent with a recent study in Tanzania, where similar rates of bacteremia (4.8% of admissions) and distribution of pathogens (*S aureus* 28%, NTS 21%, *S pneumoniae* 7%) were identified.^[7]^ Records were unavailable for nearly one-third of potentially eligible cases, and we do not know what affect this missing data had on our results.

Despite the limitations of this study, it is remarkable that there were no cases of *S pneumoniae* or Hib bacteremia. The predominance of NTS species and other enteric Gram-negatives suggests a need to re-evaluate optimal antimicrobial prophylaxis and empirical treatment in this population.^[28]^ Despite its success in resource-rich settings, penicillin prophylaxis may not be optimal for PCV and Hib-vaccinated patients with SCA in The Gambia. However, it is unclear whether possible alternatives such as co-trimoxazole or azithromycin would be safe or cost-effective. For SCA patients with suspected sepsis, empirical treatment must be effective against both NTS and *S aureus*, and account for local resistance patterns. As other countries in sub-Saharan Africa adopt PCV, they may see a changing spectrum of pathogens in SCA patients. We suggest that further research, including clinical trials, are needed to determine locally appropriate treatment and prophylaxis regimens for SCA.
